# Effects of Different Freezing Rate and Frozen Storage Temperature on the Quality of Large-Mouth Bass (*Micropterus salmoides*)

**DOI:** 10.3390/molecules28145432

**Published:** 2023-07-15

**Authors:** Yulong Bao, Yaqi Zhang, Wanjun Xu

**Affiliations:** School of Food and Biological Engineering, Jiangsu University, Zhenjiang 212013, China

**Keywords:** freezing, protein oxidation, lipid oxidation, water-holding, GC × GC-TOFMS

## Abstract

In order to clarify the individual role of freezing and frozen storage on the quality of fish, fillets of large-mouth bass (*Micropterus salmoides*) were subjected to different freezing rates (freezing with −18 °C (A), −60 °C (B), and −60 °C with forced air circulation at 2 m/s (C), respectively) followed by frozen storage at −18 °C for 30 and 90 days. Another two groups were frozen at −60 °C, followed by storage at −40 °C (D) and −60 °C (E), respectively. Results showed that water-holding and TVBN were mainly affected by storage time. No significant changes were found in free thiol content among treatments. A greater freezing rate and lower storage temperature generally led to lower TBARS. GC × GC-TOFMS revealed a total of 66 volatile compounds, which were related to lipid oxidation. PLS-DA showed that fresh samples were separated from the frozen–thawed ones, and fillets in groups D and E were relatively close to fresh fillets in the composition of oxidation-related volatiles. In conclusion, freezing rate and storage temperature had a significant impact on lipid oxidation and protein denaturation in the fillets of large-mouth bass, while protein oxidation was more affected by freezing rate.

## 1. Introduction

Large-mouth bass (*Micropterus salmoides*) is one of the important commercial fish species and is popular due to its high proportion of unsaturated fatty acids, especially EPA and DHA, and also its delicate texture and excellent taste. In recent years, the growth rate of large-mouth bass yield was one of the highest among all the freshwater fish species in China [1]. Freezing is one of the most common storage methods for fish and fish products. However, freezing of muscle foods can lead to a series of physicochemical changes such as the formation of ice crystals and the ice-water interface, concentration of pro-oxidants, altered pH and ionic strength, etc. [2]. Although frozen storage of muscle foods is a common practice, new insights into the mechanism of freeze-induced damage are still emerging [3,4]. Many studies have shown that different freezing rates and frozen storage temperatures have significant effects on fish quality indicators, including texture, water-holding, flavor, etc. [5,6]. However, it is often difficult to distinguish the individual contribution of freezing rate and storage temperatures to specific quality indicators.

Among the quality deterioration in frozen muscle, lipid oxidation is of particular importance. The large-mouth bass is generally prepared by steaming as the original taste and odor are favored by consumers. Therefore, extensive off-flavor in this kind of fish is especially unacceptable. Oxidation of polyunsaturated fatty acids and subsequent decomposition of lipid-oxidation products can produce volatiles, which lead to a fishy odor [7]. Traditionally, the oxidation of lipids has been followed by measuring TBARS and PV values. Volatiles generated from oxidizing fatty acids, such as hydrocarbons, alcohols, aldehydes, acids, etc., have been widely used to assess the extent of lipid oxidation [8]. Volatile compounds of meat are very complex, and the identification/quantification requires powerful instruments. As pointed out by Dunkel et al. [9], a total number of 10,000 volatiles was predicted to occur in foods, and omitting just one key odorant will induce significant deviations in the perception of aroma. Therefore, the analytical coverage of the chemical odor space of food needs to be comprehensive. In recent years, two-dimensional gas chromatography (GC × GC) has been applied in the analysis of food odor [10,11,12]. Its high resolution may allow the separation of target compounds from matrix interferences or coeluted substances [13].

The aim of this study was to classify the individual contribution of freezing rate and frozen storage temperature on selected quality indicators of frozen/thawed large-mouth bass, with a focus on volatiles related to lipid oxidation revealed by GC × GC-TOFMS.

## 2. Results and Discussion

### 2.1. The Freezing Process and Frozen Storage

**Figure 1:**
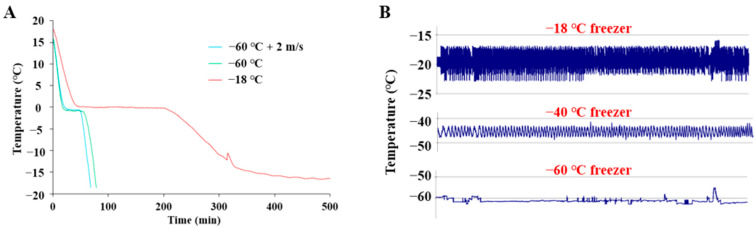
Core temperature of large-mouth bass fillets during freezing until the center reached −18 °C (**A**), and typical temperature fluctuations within different freezers over a period of 1 month; the temperature was recorded every 15 min (**B**).

The core temperatures of fillets subjected to different freezing treatments were recorded. As the freezing process started, the temperature gradually dropped. Then, the temperature reached a plateau, and the temperature corresponding to the plateau was lower when freezing at −60 °C compared to freezing at −18 °C (Figure 1A). Similarly, the phenomenon that faster freezing resulted in a relatively lower temperature at the plateau was also observed in the freezing of muscle foods [14,15] and other non-muscle food [16]. This difference may have resulted from different freezing rates. It is well-known that during slow freezing, the formation of ice will release the latent heat of the water and thereby obtain heat removal by cooling. As a result, the system maintained a rather constant temperature. When the cooling rate was higher, the heat removal would be greater than the released latent heat; therefore, the equilibrium temperature would be lower. At an even higher freezing rate, such as freezing with liquid nitrogen, a plateau temperature is hardly seen. It can be seen that freezing at −60 °C was much faster than freezing at −18 °C. The time needed for the temperature to decrease from −1 °C to −5 °C (maximal ice formation region) was approximately 47 min when freezing at −18 °C, while it took 13 min at −60 °C. The additional forced air circulation at 2 m/s further reduced that time to 7 min. Throughout the storage period, temperatures of the freezers were relatively stable with minor fluctuations (Figure 1B).

### 2.2. Water-Holding

**Figure 2:**
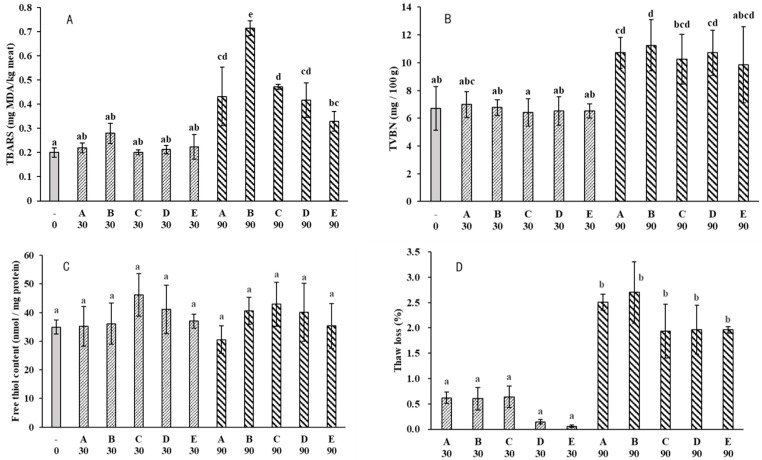

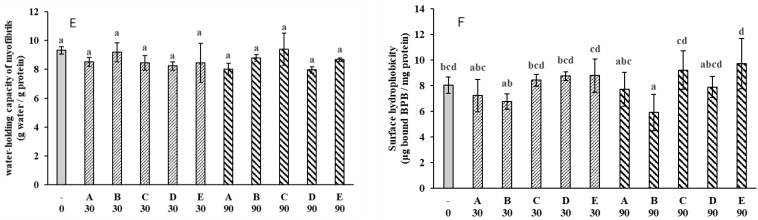
Quality indicators of large-mouth bass fillets (Subfigures: (**A**), TBARS; (**B**), TVBN; (**C**), free thiol content; (**D**), thaw loss; (**E**), water-holding capacity of myofibrils; (**F**), surface hydrophobicity). In the axis: “-”, fresh sample; Group A, freeze at −18 °C and storage at −18 °C; Group B, freeze at −60 °C and storage at −18 °C; Group C, freeze at −60 °C with forced air circulation (wind speed 2 m/s) and storage at −18 °C; Group D, freeze at −60 °C and storage at −40 °C; Group E, freeze at −60 °C and storage at −60 °C, and 0, 30, or 90 indicates the storage time in days. For each quality indicator, values sharing the same lowercase letters do not differ (*p* < 0.05).

In this study, water-holding was studied by thaw loss and the centrifugation loss of myofibrils. Results showed that only storage time had a significant effect (*p* < 0.001) on thaw loss (Table 1). Thaw loss at day 90 was more than doubled compared to day 30 (Figure 2D), while the water-holding of myofibrils did not show significant changes throughout the frozen storage (Figure 2E). This was probably due to the different principles of the two methods. Thaw loss mainly reflects the loss of extracellular water, while the centrifugation loss of myofibrils reflects water loss in the intracellular matrix. The thaw loss of fish fillets was below 3.5%, while the thaw loss of pork or beef was often greater. This could be linked to a higher ultimate pH in fish, and higher pH is often associated with better water-holding [17]. Water-holding had a close relationship with meat color, in addition to meat pigments. Disruption of the sarcolemma and the shrinkage of myofibril led to changes in water-holding, and the structural changes would affect the light-scattering properties of meat [18]. Thawed fillets had higher values of L* (lightness) compared to unfrozen fresh samples (Supplementary File S1 and Figure S2), suggesting reduced water-holding. Positive correlations between thaw loss and lightness were also reported in other freeze–thawed fish fillets [19,20]. As for the water-holding of myofibrils, two-way ANOVA revealed a significant effect of storage temperature and the interaction between freezing rate and storage time (*p* < 0.05).

### 2.3. Microstructure Changes

**Figure 3:**
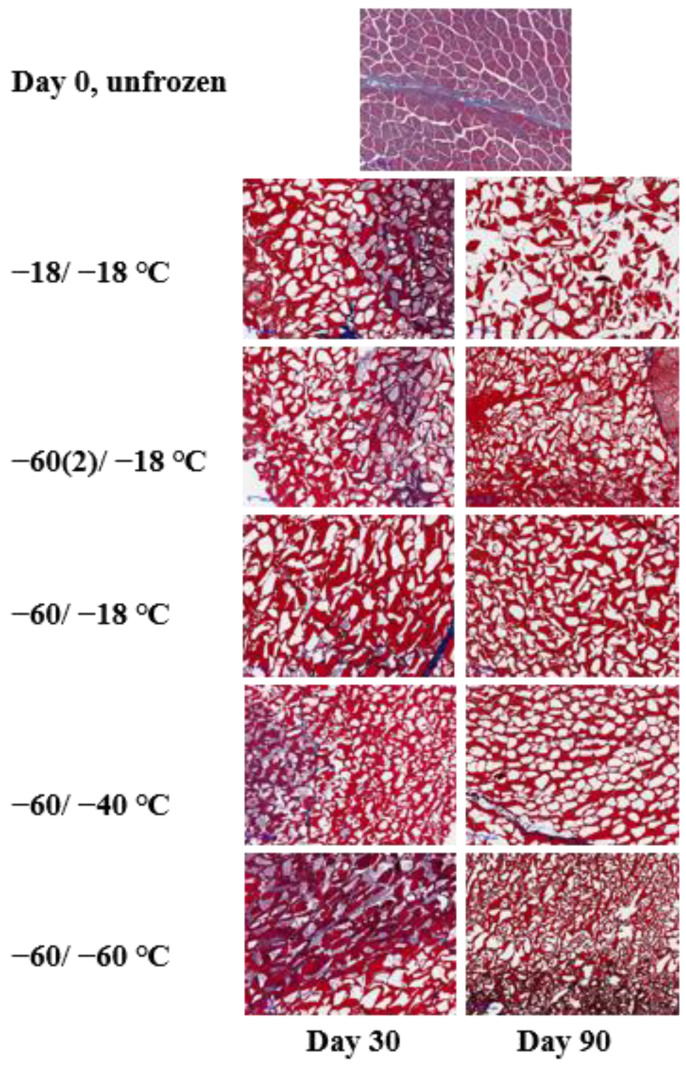
Micrograph of large-mouth bass fillets.

The combined effects of freezing rates, frozen storage, and thawing rates on crystal formation and the ultrastructure of muscle is a complex system [21]. Upon thawing, the meat structure appeared to have almost completely recovered. In order to observe the ice crystals in the frozen state, a cross-sectional micrograph of fish muscle was obtained via the freeze substitution histological observation (Figure 3). Unfrozen fresh muscle showed clear perimysium, the myofiber remained intact, and the extracellular space was relatively small. The formation of ice crystals and distortion of myofibers can be observed in all frozen samples. It has been suggested that the location and geometry of ice crystals can cause physical damage to muscle microstructure and alter the composition of the unfrozen fraction, which would affect the biochemical reactions in frozen meat [2]. As shown in Figure 1B, the temperature fluctuation in the −18 °C freezer was around ±3 °C; this temperature fluctuation led to ice recrystallization. As demonstrated by Bevilacqua and Zaritzky [22], ice crystals in frozen muscle can undergo significant recrystallization within a short period of storage at −5 °C, with an average diameter increased from 15 μm to 30 μm at day four. Generally, the ice crystals became larger at day 90 compared to day 30 due to recrystallization during frozen storage. The larger ice crystals likely contributed to a larger thaw loss at day 90, due to the physical damage of muscle structure rather than protein denaturation, since protein denaturation, when evaluated by surface hydrophobicity, did not change much from day 30 to day 90. Samples that were frozen at −18 °C and stored at −18 °C for 90 days displayed the most severe damage to muscle structure. However, the structural damage was comparable to other groups at day 30, suggesting that relatively long-term frozen storage at −18 °C was more detrimental to muscle structure.

### 2.4. Lipid Oxidation

Lipid oxidation can progress to a noticeable extent during the long-term frozen storage of meat products. It can be seen that the lipid oxidation level in the fillets measured as TBARS was not significantly different after frozen storage for 30 days, regardless of the freezing/storage treatment (Figure 2A). However, TBARS values all increased at day 90 compared to the day 0 fresh sample. The increase of TBARS in frozen muscle foods with time has been widely reported [23,24,25,26]. Long storage led to a greater extent of the release of heme-iron, which may catalyze lipid oxidation [27]. The combination of different freezing rates and frozen storage temperatures also had a significant effect on TBARS. At day 90, fillets that were frozen at −60 °C and stored at −18 °C (Group B) showed the highest TBARS value, around 0.7 mg MDA/kg meat. Group B had the same freezing rate as groups D and E, but subsequent frozen storage at −18 °C increased lipid oxidation. According to Domínguez et al. [8], processes that cause disruption of sarcolemma will liberate membrane phospholipids and increase contact with pro-oxidants. The higher TBARS value in group B may have resulted from a greater disruption of sarcolemma due to more severe ice recrystallization (Figure 3).

It is well established that the freezing rate affects the location and morphology of ice crystals in frozen meat [28]. In very fast freezing, small ice crystals mainly form intracellularly, while a slow freezing rate mainly produces extracellular ice crystals. In both cases, the physical damage to sarcolemma by ice crystal was expected to be lower than freezing at a medium rate, where larger crystals form intracellularly. In agreement, Group A and C differ in freezing rate compared to Group B, which had lower TABRS values [29]. It was suggested that the distribution of tissue salts within the frozen muscle is another key factor affecting biochemical reactions. In rapidly frozen meat, cell solutes became occluded in the ice, thus keeping from contact with cell components such as the sarcolemma. During the subsequent frozen storage, ice recrystallization would lead to the liberation of the occluded material and contact with cell components. This may be another reason why group B had higher TBARS than groups D and E.

Two-way ANOVA analysis revealed that storage time, freezing rate, and frozen storage temperature all had significant effects on TBARS (*p* < 0.001), and there was a significant interaction of freezing rate ∗ storage time and storage temperature ∗ storage time (*p* < 0.01) (Table 1). Similarly, Hou et al. [24] reported that freezing methods (with different freezing rates) had a significant effect on the TBARS value of pork, and there was a strong interaction between the freezing rate and storage time. Storage at −80 °C for 20 weeks did not increase the TBARS value in beef. Karlsdottir et al. [30] studied the effects of temperature during frozen storage on lipid deterioration of saithe and hoki muscles; results showed that extended storage time increased lipid deterioration, but lower storage temperature had more preservative effects. One limitation of TBARS measurement is that it does not provide enough information regarding the undergone oxidation process; other measurements such as peroxide value, conjugated diene value, p-anisidine value, free fatty acids, or total polar compounds should be considered in further investigations.

It is known that lipid oxidation generates a range of small molecular compounds, including aldehydes, ketones, alcohols, and acids, and many of them are volatile [31]. Simultaneous detection and quantification of volatiles provide us with more realistic and complete information [8]. Baron [23] concluded that the measurement of volatiles is a very sensitive method for measuring the development of lipid oxidation in rainbow trout during frozen storage at different temperatures. In order to study lipid oxidation in more detail, GC × GC-TOFMS was employed to investigate possible products degraded from lipid oxidation, and only the compound presented in at least three replicates was considered reliable. In total, 40 compounds were quantified and presented in Supplementary File S2. In general, the volatile profile may be considered a chemical fingerprint of food: the nature and relative quantities of compounds in the volatile fraction are distinctive features. Analyzing volatile compounds can be exploited to differentiate fresh fish from frozen–thawed ones. Leduc et al. [32] compared the volatile compounds both in fresh and frozen–thawed European sea bass, gilthead seabream, cod, and salmon, and results showed that dimethyl sulfide, 3-methybutanal, ethyl acetate, and 2-methybutanal were suitable differentiation indicators. Zhang et al. [33] suggested using a volatile profile to evaluate the freshness of different seafood; as a conventional chemical marker, trimethylamine does not always have a high contribution to discrimination.

**Figure 4:**
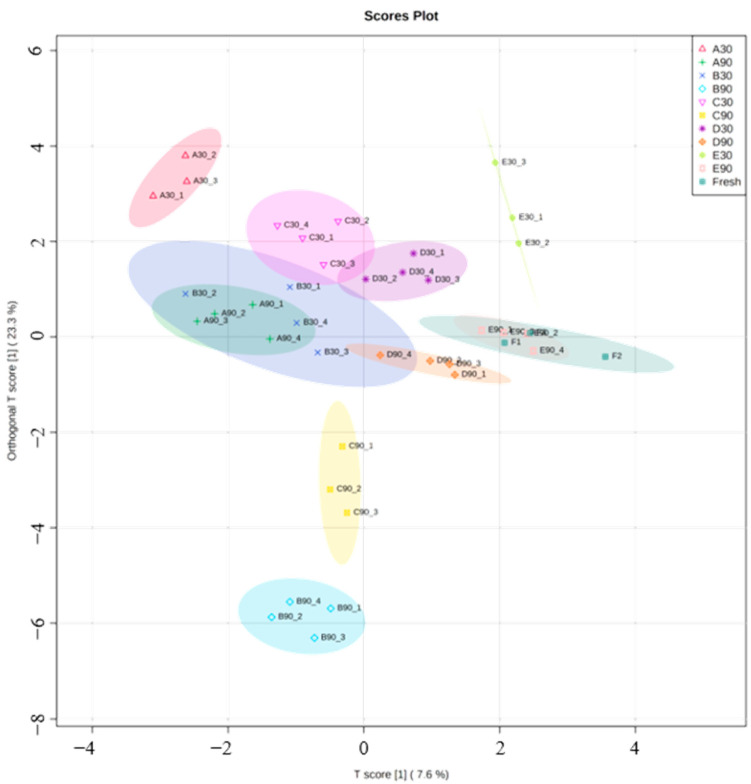
OPLS-DA of the volatiles in large-mouth bass fillets related to lipid oxidation. Group A, freeze at −18 °C and storage at −18 °C; Group B, freeze at −60 °C and storage at −18 °C; Group C, freeze at −60 °C with forced air circulation (wind speed 2 m/s) and storage at −18 °C; Group D, freeze at −60 °C and storage at −40 °C; Group E, freeze at −60 °C and storage at −60 °C, and 30 or 90 indicates the storage time in days.

In the present study, a range of aldehydes (2-octenal, 2-nonenal, etc.), ketones (2-undecanone, 2-nonanone, etc.), alcohols (3-octanol, 2-octen-1-ol, etc.), and acids (hexanoic acid, heptanoic acid, etc.) was only presented in frozen–thawed fillets. This likely contributed to our observation that fresh samples were separated from the frozen–thawed ones except for E90 in OPLS-DA (Figure 4). For samples taken on day 30 and day 90, ANOVA revealed that storage time, storage temperature, and the freezing rate before storage all had significant effects on volatile composition (Table 2). Different volatiles responded to frozen storage conditions differently. Among the 40 quantified compounds, 2-pentanone, 1-pentanol, 1-hexanol, 1-heptanol, and (5z)-octa-1,5-dien-3-ol were significantly affected by all three factors, while octanoic acid, 3-phenyl-2-propenal, n-decanoic acid, octanal, 6-methyl-5-hepten-2-one, 6-methyl-3-heptanol, nonanal, 2-octenal, and 2-nonenal were not affected by either of the three factors. From the OPLS-DA graph (Figure 4), it can be found that fillets in groups D and E were relatively close to fresh fillets in the composition of oxidation-related volatiles, suggesting that frozen storage at temperatures below −40 °C was beneficial in preserving the freshness of large-mouth bass in terms of volatiles. In agreement with the TBARS test, group B at day 90 had a higher content of oxidation-derived compounds, such as 1-pentanol and 1-octen-3-ol, compared to other treatments. In addition, a range of oxidation-derived compounds was only detected at day 90 in group B, including heptanal, 2-hexenal, 2,4-heptadienal, 4-hepten-1-ol, 2,7-octadien-1-ol, and undecanal. According to Barriuso et al. [34], some volatile compounds are highly specific to the oxidation of particular fatty acids. The pathway that led to the complex profile of the volatiles requires further research. The preliminary results obtained in this study showed that the application of GC × GC-TOFMS in the study of frozen fish quality is feasible. 

### 2.5. Protein Changes

Protein denaturation and oxidation are common consequences of frozen–thawed muscle foods, and both phenomena affect frozen–thawed meat quality, such as water-holding. Protein denaturation and oxidation are likely to be closely related [2]. In this study, protein denaturation and oxidation were indicated by increased surface hydrophobicity and loss of free thiols, respectively. In general, there were no marked changes in protein oxidation when evaluated by free thiols (Figure 2), but two-way ANOVA revealed that the freezing rate had a significant effect (*p* < 0.01, Table 1). Similarly, Hou et al. [24] reported that free thiols did not change between slow-freezing and fast-freezing pork, but lipid oxidation was lower in fast-freezing samples. In theory, oxidative stress can be transferred between lipids and proteins. In this study, lipid oxidation displayed significant changes between storage time and freezing treatments. Many studies observed increased lipid oxidation in frozen meats together with increased protein oxidation [35,36,37]. As for protein denaturation, there were significant changes in surface hydrophobicity of extracted myofibrils among freezing treatments, and both freezing rate and storage temperature exhibited a strong effect (Table 1). In agreement, Zhang and Ertbjerg [4] reported that slow freezing led to increased surface hydrophobicity of myofibrils. Qian et al. [38] found that protein denaturation was generally greater at higher temperatures when beef was frozen and stored at temperatures between −1 and −18 °C. Proteolytic activity may proceed during frozen storage, suggested by an increase of TVB-N with storage time. TVB-N is widely used as an indicator of fish freshness and results from the degradation of protein and non-protein nitrogenous compounds by microbial and endogenous enzymes. Neither the storage temperature nor the freezing rate had a significant effect on the amount of TVB-N (*p* > 0.05, Table 1).

## 3. Materials and Methods

### 3.1. Sample Preparation

Fresh large-mouth bass (*Micropterus salmoides*) were purchased from a local supermarket (Wuxi, China) in August. Those fish were stunned at the head with a wooden stick, followed by the removal of scales and internal organs. The fish buried in crushed ice were transported to the lab within half an hour. Upon arrival, each fish was rinsed under tap water, and their weight was recorded (weight: 405 ± 34 g). The dorsal muscle was taken out and cut into fillets of 5 cm ∗ 3.5 cm. The fillets were divided into five groups and subjected to different freezing/frozen storage treatments. Each fillet was frozen by cold air in a freezer till the center of the fillet reached a temperature of −18 °C (monitored by a K-type thermocouple), and then, the fillet was transferred to frozen storage at different temperatures. The treatment was as follows: Group A, freeze at −18 °C and storage at −18 °C; Group B, freeze at −60 °C and storage at −18 °C; Group C, freeze at −60 °C with forced air circulation (wind speed 2 m/s) and storage at −18 °C; Group D, freeze at −60 °C and storage at −40 °C; Group E, freeze at −60 °C and storage at −60 °C. The groups were designed to evaluate the effects of different freezing rates (Groups A/B/C) and different frozen storage temperatures (Groups B/D/E). The temperature of the freezers during frozen storage was recorded by a K-type thermocouple (Uni-Trend Technology, Dongguan, China). After frozen storage for 30 and 90 days, the fillets were sampled for physiochemical analysis. Fresh fillets were used as control.

### 3.2. Water-Holding Capacity

Water-holding capacity was evaluated by thaw loss and centrifugation loss. Frozen fillets were thawed at room temperature. Each frozen fillet and the corresponding thawed fillet were weighed and recorded as M_1_ and M_2_, respectively. The thaw loss was calculated by the formula: Thaw loss (%) = (M_1_ − M_2_)/M_1_ ∗ 100. For centrifugation loss, myofibrils were extracted from thawed fillets, and the water-holding capacity of myofibrils was determined, as described by Ji et al. [14]. Briefly, 1 mL of myofibrils was centrifuged at 2400× *g* for 10 min, and the weight of the wet myofibrils was recorded as W_1_. The wet myofibrils were dried, the dry weight was recorded as W_2_, and the water-holding of myofibrils was calculated as W_1_/W_2_ g H_2_O/g protein.

### 3.3. Lipid Oxidation

The thiobarbituric acid-reactive substance (TBARS) test was used to estimate the degree of lipid oxidation based on Bao et al. [39] with some modifications. Briefly, minced fish muscle (5 g) was homogenized (IKA T18 Ultra-Turrax, Labortechnik, Staufen, Germany) with 20 mL of 5% trichloroacetic acid at a speed of 14,000 rpm for 20 s and then the mixture was filtered through double layers of filter paper. An aliquot of 5 mL filtrate was mixed with 5 mL thiobarbituric acid (20 mM) and incubated in a 90 °C water bath for 30 min. After cooling to room temperature, the absorbance at 532 nm was measured. Tetraethoxypropane was used as the standard because it forms equal moles of malonaldehyde (MDA) during heating in acid conditions. TBARS values were expressed as mg MDA/kg meat.

### 3.4. Total Volatile Basic Nitrogen (TVB-N)

TVB-N values were estimated according to the current Chinese standard method (method III, micro diffusion) to determine TVB-N in foods (GB5009.228-2016). Briefly, minced fish muscle (5 g) was homogenized (13,500 rpm, 20 s) with 25 mL cold distilled water, and the mixture was incubated at room temperature for 30 min, followed by filtration. An aliquot of 1 mL filtrate was added to the outer chamber of a micro-diffusion dish (Supplementary File S1 and Figure S1), and 1 mL of boric acid (20 g/L) with methyl red-bromocresol green mixed indicator was added to the inner chamber of the diffusion dish. After the addition of 1 mL of saturated K_2_CO_3_ solution to mix with the filtrate, the diffusion dish was sealed and incubated at 37 °C for 2 h. Then, the boric acid solution in the inner chamber was titrated with 0.01 M HCl; the TVB-N value was expressed as mg nitrogen/100 g muscle.

### 3.5. Protein Oxidation and Denaturation

The surface hydrophobicity of myofibrils was used as an indicator of protein denaturation. It was evaluated based on the method proposed by Chelh et al. [40]. This method was based on the interaction of the hydrophobic chromophore bromophenol blue (BPB) with myofibrillar proteins and the separation of free and bound BPB. Some modifications were made as described in Ji et al. [14]. Protein oxidation was indicated by the loss of free thiol groups, and it was determined according to Bao et al. [39].

### 3.6. Freeze Substitution Histological Observation

Prior to analysis, meat samples were kept at −18 °C overnight and then cut into small cubes (5 × 5 × 3 mm^3^). The unthawed meat cubes were fixed in Carnoy solution (60% ethanol, 30% chloroform, and 10% acetic acid) at −18 °C overnight. After fixation, the samples were dehydrated at 4 °C with a graded series of ethanol solutions (70–90% in 5% increments). Then, the samples were further processed as described in Bao et al. [41] until micrographs were taken by a light microscope (DM 2000, Leica Microsystems CMS GmbH).

### 3.7. Comprehensive Two-Dimensional Gas Chromatography Time-of-Flight Mass Spectrometry (GC × GC-TOFMS)

Samples from fresh and thawed fillets (four samples per group) were subjected to GC × GC-TOFMS analysis. Two grams of minced meat was mixed with 10 μL of internal standard (1,2-dichlorobenzene, 50 mg/L) and transferred to a 20 mL glass GC vial. The instrumental conditions were set up according to Wang et al. [42]. Briefly, a Pegasus^®^ 4D GC × GC-TOFMS system (LECO Corp., St. Joseph, MI, USA) equipped with an Agilent 7890B GC, a secondary oven, and a dual-stage quad-jet thermal modulator was used. A 60 m × 0.25 mm × 0.25 µm DB-FFAP (Agilent Technologies, Palo Alto, CA, USA) was used as the first-dimensional column, and a 1.5 m × 0.25 mm × 0.25 µm Rxi-17Sil MS (Restek, Bellefonte, PA, USA) was used as the second column. The initial oven temperature was 45 °C and kept for 3 min, raised to 150 °C at 4 °C/min and held for 2 min, then ramped at 6 °C/min to 200 °C and at 10 °C/min to 230 °C for 20 min. The modulation period of 4 s and hot pulse of 0.80 s was performed. The second oven was kept 5 °C higher than the first oven. The electron energy of TOFMS (*m*/*z* 35 to *m*/*z* 400) was −70 volts, and the acquisition rate was 100 spectra/s; the ion source and transfer line were 230 °C and 240 °C, respectively.

The data were processed with ChromaTOF version 4.61.1.0 software (LECO Corp., St. Joseph, MI, USA). The peaks aligned by the software are based on the extracted masses and their retention times (first- and second-dimensional). Identification was done by comparing mass spectra and retention times (first- and second-dimensional) in databases (NIST 2014 and Welly 9). Identified compounds with similarity values larger than 800 were considered, and only the compound that was present in at least three replicates was considered reliable.

### 3.8. Data Analysis

Analysis of variance (ANOVA) with a post-hoc test using the Duncan method (*p* < 0.05) was performed by the SPSS 22 statistics package program (IBM Corp., New York, NY, USA). For multi-factor ANOVA, day 30 and day 90 samples were used. OPLS-DA analysis of volatiles was performed with MetaboAnalyst 4.0 (https://www.metaboanalyst.ca/ (accessed on 15 December 2022)). Data processing methods: data transformation: cubic root transformation; data scaling: Pareto scaling. The 2D score plot of PLS-DA displayed a 95% confidence region.

## 4. Conclusions

The present study found that freezing rate and storage temperature had a significant impact on lipid oxidation and protein denaturation in fillets of large-mouth bass, while protein oxidation was more affected by freezing rate. Water-holding and TVBN were mainly affected by storage time. GC × GC-TOFMS revealed a total of 40 volatile compounds, which may derive from lipid oxidation and degradation. OPLS-DA of the detected volatiles showed that fresh samples were separated from frozen–thawed ones. Volatiles such as 2-octenal, 2-nonenal, 2-undecanone, 2-nonanone, 2-octen-1-ol, hexanoic acid, and heptanoic acid were only presented in frozen–thawed fillets. Fillets that were frozen at −60 °C with forced air circulation at 2 m/s were generally separated from samples frozen at −18 °C or −60 °C (without forced air circulation), and fillets that were frozen and stored at −40 °C and −60 °C were relatively close to fresh fillets in the composition of oxidation-related volatiles, indicating that a faster freezing rate and storage at temperatures below −40 °C were beneficial in preserving freshness of large-mouth bass in terms of volatiles. However, the level of temperature may have some limitations regarding an industrial implementation of these conditions. In addition, the chemical composition of fillets of large-mouth bass before and after the application of different freezing/storage treatments could be performed in future studies. Another interesting but challenging measurement is the compositional changes of the unfrozen fractions, which would provide a theoretical interpretation of the observed results.

## Figures and Tables

**Table 1:** Main effects of storage time (t), freezing rate (FR), and storage temperature (ST) and their interaction on selected quality indicators of frozen–thawed large-mouth bass fillets.

Indicator	Time t	Freezing Rate FR	Temperature ST	t ∗ FR	t ∗ ST
**TVBN**	***	NS	NS	NS	NS
**TBARS**	***	***	***	**	***
**Water-holding of myofibrils**	NS	NS	*	*	NS
**Thaw loss**	***	NS	NS	NS	NS
**Thiols**	NS	**	NS	NS	NS
**Surface hydrophobicity**	NS	***	***	NS	NS

NS, not significant; *, *p* < 0.05; **, *p* < 0.01; ***, *p* < 0.001.

**Table 2:** Main effects of freezing rate, storage temperature, and storage time on lipid-oxidation related compounds of frozen–thawed large-mouth bass fillets as detected by GC × GC-TOFMS.

Compounds	Freezing Rate	Storage Temperature	Storage Time
**Hexanoic acid**	NS	NS	**
**Heptanoic acid**	NS	NS	*
**Pentadecanal**	*	NS	NS
**Octanoic acid**	NS	NS	NS
**2-Propenal, 3-phenyl-**	NS	NS	NS
**Nonanoic acid**	NS	NS	**
**n-Decanoic acid**	NS	NS	NS
**Heptanal**	***	***	NS
**2-Hexenal**	***	***	NS
**2-Pentanone**	***	***	***
**1-Pentanol**	***	***	***
**1-Heptadecanol**	NS	***	***
**2-Butanone, 3-methyl-**	NS	***	**
**2-Butanone, 3-hydroxy-**	NS	**	*
**Octanal**	NS	NS	NS
**2,3-Octanedione**	*	*	NS
**5-Hepten-2-one, 6-methyl-**	NS	NS	NS
**1-Hexanol**	***	***	**
**3-Heptanol, 6-methyl-**	NS	NS	NS
**2-Nonanone**	NS	NS	*
**Nonanal**	NS	NS	NS
**2-Octenal**	NS	NS	NS
**1-Octen-3-ol**	*	*	NS
**1-Heptanol**	***	***	**
**Decanal**	NS	**	*
**2,4-Heptadienal**	***	***	NS
**(5Z)-Octa-1,5-dien-3-ol**	***	***	**
**4-Hepten-1-ol**	***	***	NS
**1-Hexanol, 2-ethyl-**	NS	NS	*
**2-Nonenal**	NS	NS	NS
**1-Octanol**	NS	NS	*
**2,7-Octadien-1-ol**	***	***	NS
**Undecanal**	***	***	NS
**3-Undecanone**	***	NS	**
**2-Undecanone**	NS	*	NS
**2-Octen-1-ol**	**	NS	NS
**2-Undecanone**	NS	*	NS
**1-Nonanol**	NS	*	*
**11-Dodecenol**	***	***	NS
**2(5H)-furanone**	NS	NS	*

NS, not significant; *, *p* < 0.05; **, *p* < 0.01; ***, *p* < 0.001.

## Data Availability

Data will be available upon reasonable request.

## Supplementary Materials

The following supporting information can be downloaded at: https://www.mdpi.com/article/10.3390/molecules28145432/s1, Supplementary File S1: TVB-N measurement device and the Lightness of large-mouth fillets. Figure S1: Illustration of the micro-diffusion dish used for the measurement of TVB-N; Figure S2: Lightness (L*) of large-mouth fillet subjected to different freezing/frozen storage treatments. Supplementary File S2: summary of quantified volatiles in the large-mouth fillet subjected to different freezing/frozen storage treatments.
