# Supplementary material for: Effects of Different Freezing Rate and Frozen Storage Temperature on the Quality of Large-Mouth Bass (Micropterus salmoides)
Source: Molecules. 2023 Jul 15;28(14):5432. doi: 10.3390/molecules28145432 (PMC10385098; doi:10.3390/molecules28145432)
Supplement: Supplementary file 1 [file molecules-28-05432-s001.zip › Supplementary File S1.pdf]

Supplementary Materials

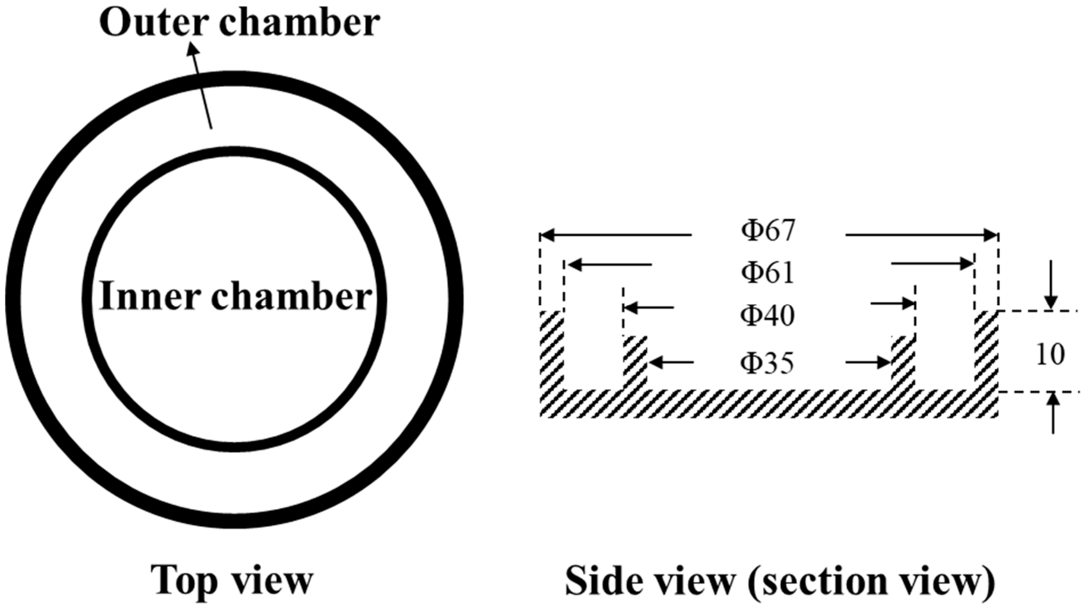

Supplementary Figure S1. Illustration of the micro-diffusion dish.

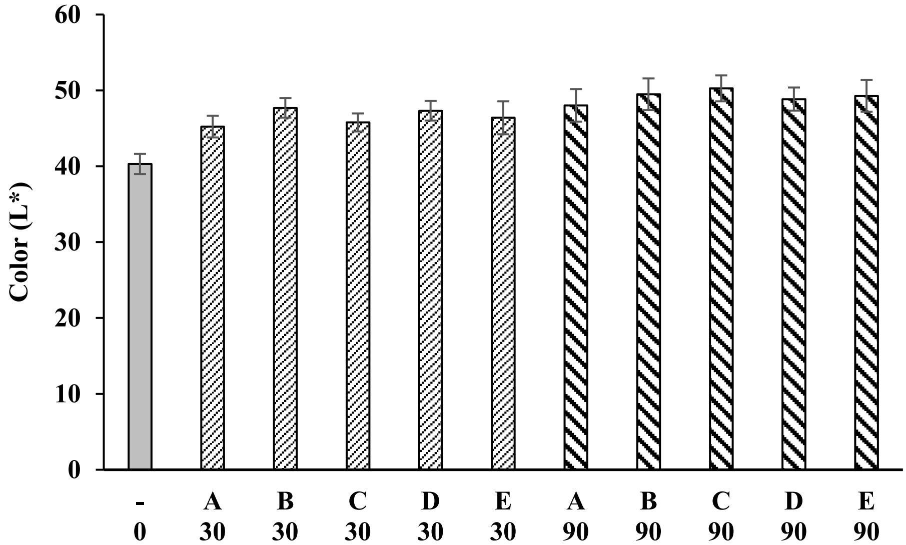

Supplementary Figure S2. Lightness (L*) of large-mouth fillet subjected to different freezing/frozen storage treatments. "-", fresh sample; Group A, Freeze at -18 °C and storage at -18 °C; Group B, Freeze at -60 °C and storage at -18 °C; Group C, Freeze at -60 °C with forced air circulation (wind speed 2 m/s) and storage at -18 °C; Group D, Freeze at -60 °C and storage at -40 °C; Group E, Freeze at -60 °C and storage at -60 °C.
